# Efficiency of sweet whey fermentation with psychrophilic methanogens

**DOI:** 10.1007/s11356-021-14095-y

**Published:** 2021-05-02

**Authors:** Marcin Dębowski, Ewa Korzeniewska, Joanna Kazimierowicz, Marcin Zieliński

**Affiliations:** 1 Department of Environment Engineering, Faculty of Geoengineering, University of Warmia and Mazury in Olsztyn, 10-720 Olsztyn, Poland; 2 Department of Water Protection Engineering and Environmental Microbiology, Faculty of Geoengineering, University of Warmia and Mazury in Olsztyn, 10-720 Olsztyn, Poland; 3 Department of Water Supply and Sewage Systems, Faculty of Civil Engineering and Environmental Sciences, Bialystok University of Technology, 15-351, Bialystok, Poland

**Keywords:** Methane fermentation, Psychrophilic methanogens, Sweet whey, Biogas

## Abstract

Sweet whey is a waste product from the dairy industry that is difficult to manage. High hopes are fostered regarding its neutralization in the methane fermentation. An economically viable alternative to a typical mesophilic fermentation seems to be the process involving psychrophilic bacteria isolated from the natural environment. This study aimed to determine the feasibility of exploiting psychrophilic microorganisms in methane fermentation of sweet whey. The experiments were carried out under dynamic conditions using Bio Flo 310 type flow-through anaerobic bioreactors. The temperature inside the reactors was 10 ± 1 °C. The HRT was 20 days and the OLR was 0.2 g COD/dm^3^/day. The study yielded 132.7 ± 13.8 mL biogas/g_CODremoved_. The CH_4_ concentration in the biogas was 32.7 ± 1.6%, that of H_2_ was 8.7 ± 4.7%, whereas that of CO_2_ reached 58.42 ± 2.47%. Other gases were also determined, though in lower concentrations. The COD and BOD_5_ removal efficiency reached 21.4 ± 0.6% and 17.6 ± 1.0%, respectively.

## Introduction

The production processes carried out in dairy plants generate waste and sewage, which pose a threat to the natural environment (Sehar and Nasser 2019; Żyłka et al. 2020). One of the main by-products troublesome in management is sweet whey produced in the cheese-making process (Rocha and Guerra 2020). It is usually re-used in the food industry, e.g., to produce special-purpose dietary foods and high-protein supplements (Slozhenkina et al. 2020). The most popular whey processing methods include lactose removal by nanofiltration, demineralization via ionic exchange, or demineralization by electrodialysis, fractionation by membrane filtration, isolation of components by ionic exchange, concentration, evaporation, or drying (Wen-Qiong et al. 2019; Smykov 2020).

Because typical sweet whey processing methods are often expensive, technologically complex, and not always possible to be implemented, a search is underway for competitive solutions enabling economically and ecologically justified waste management (Ritambhara et al. 2019). In this case, a prospective technology seems to be methane fermentation, the main products of which include biogas containing significant amounts of methane and a stable fertilizer rich in humic and biogenic substances (Koniuszewska et al. 2020; Wiater et al. 2019). However, the common use of methane fermentation under mesophilic conditions is curbed by the high sensitivity of microorganisms to fluctuations in environmental factors and the costs associated with, among others, heating bioreactors (Sarker et al. 2019). While at thermophilic conditions, frequent acidification incidents were recorded, the limited buffer capacity of the influent feedstock was responsible for the unstable anaerobic digestion process (Treu et al. 2018). Innovative techniques exploiting the high enzymatic activity of selected strains of psychrophilic and psychrotrophic microorganisms offer an alternative in this respect (Yao et al. 2020). Their implementation would enable a significant reduction in the operating expenses incurred to maintain appropriate thermal conditions in the reactors and allow disseminating anaerobic technologies in regions with unfavorable climatic characteristics.

During biogas production, the reactor efficiency can be increased through an accurate monitoring of process parameters, including reactor characteristics, temperature, mixing, feedstock composition (Angelidaki et al. 2018). All these factors can have a direct effect on the microbial community (Zhu et al. 2019). However, despite the microbiome’s pivotal role in organic matter conversion into methane, there is still a lack of knowledge regarding the microbial influence on the process (Koch et al. 2019). During biogas production, when acetoclastic methanogenic archaea are inhibited, a pivotal role is played by hydrogenotrophic methanogenic archaea and syntrophic acetate-oxidizing bacteria (Mosbæk et al. 2016) (SAOB). An example is the mutualism between the hydrogen-utilizing methanogen *Methanoculleus bourgensis* and the SAOB *Syntrophaceticus schinkii*, *[Clostridium] ultunense*, and *Tepidanaerobacter acetatoxydans* (Westerholm et al. 2019). SAOB oxidize acetate to formate or to H_2_ and CO_2_. The bacteria rely on archaeal activity, because acetate oxidation rapidly becomes endergonic when H_2_ accumulates. Indeed, subsequently, H_2_-utilizing methanogens convert these substrates to CH_4_ (Treu et al. 2018). Ammonia is the only type of a methane fermentation inhibitor for which the literature provides information on the impact on the activity of microorganisms involved in the process, as well as changes in the structure of their population. In the case of the other inhibitory compounds, these data are very scarce and require verification (ions of light metals, heavy metals, antibiotics, ethylene and acetylene, chlorophenols), or the literature does not provide any information about them (sulfides, halogen aliphatic hydrocarbons, aliphatic nitro compounds, long-chain fatty acids). Therefore, more research is required in order to identify the influence of inhibitory and toxic substances on the activity of methane fermentation microbiota, which will allow us to ensure the optimal conditions for the growth and development of these microorganisms. Such research should rely on some modern research tools, for example, NGS sequencing (Czatzkowska et al. 2020).

The findings from the research on psychrophilic fermentation are considered prospective (Gunes et al. 2019). In-storage psychrophilic anaerobic digestion (ISPAD) (Giard et al. 2013) and the stabilization of sewage sludge under psychrophilic fermentation in the digestion chambers (Pilarski et al. 2019) are used. The effectiveness of commercial-scale psychrophilic anaerobic digestion in sequencing batch reactors (PADSBRs) for pathogen removal from pig manure (Massé et al. 2011) has been demonstrated. The drawbacks highlighted by the researchers include the long period of the anaerobic microflora adaptation to low temperatures and the required long hydraulic retention time (HRT) of the substrates, which significantly increases the volume of fermenters (Dev et al. 2019). Therefore, it is necessary to look for such solutions that would be satisfactory from both the technological and economic point of view. The efficiency of psychrophilic fermentation can be improved by learning the mechanisms of biogas production by psychrophilic microorganisms isolated from natural ecosystems (Yao et al. 2020), because an average annual temperature of over 85% of the Earth’s biosphere is below 5 °C (Rivkina et al. 2004).

Methanogenic microorganisms, which are strict anaerobes, are widespread in the natural environment, including, i.e., bottom sediments of surface reservoirs (Tabassum and Rajoka 2000), peat bogs (Garcia et al. 2000), and the digestive system of humans and animals (Levitt et al. 2006). They also colonize arctic regions, where they are capable of not only surviving and growing relatively quickly at temperatures below − 20 °C, but also carrying out metabolic reactions at temperatures close to 0 °C (Gilichinsky 2004; Rivkina et al. 2004). Temperature affects microorganisms directly by influencing their growth rate, enzyme activity, cell composition and nutritional requirements, or indirectly by regulating the solubility of intracellular molecules, ionic transport and diffusion, and modifying the osmotic properties of cell membranes (Choudhary et al. 2020). The resistance of psychrophilic and psychrotrophic microorganisms to low temperatures is due to mutations in genes encoding for both ribosomal and enzyme proteins. Microorganisms adapted to living in low temperatures secrete enzymes that work effectively at temperatures typical of the natural environment colonized by these microorganisms (Adler and Knowles 1995).

This study aimed to determine the feasibility of using psychrophilic and psychrotrophic microorganisms in the methane fermentation of sweet whey and assess the technological outcomes of the process, including contaminant removal, biogas production, and biogas qualitative composition.

## Methods

### Materials

The fermentative psychrophiles used in the experiments were isolated from above-bottom waters of inland reservoirs, from a depth of 22–34 me. A cocktail of strains used included *Serratia plymuthica*, *Serratia proteamaculans*, *Serratia liquefaciens, Rahnella aquatilis*, *Clostridium algidixylandycum*, *Rahnella aquatilis*, *Carnobacterium maltaromaticum*, *Trichococcus collinsii*, *Methanococcoides burtonii*, and *Methanogenium frigidum*. They were isolated onto the Brucella agar (with the addition of defibrinated blood, hemin, and vitamin K), the Brewer Anaerobic agar, and a liquid thioglycollate medium, and incubated under anaerobic conditions in a BACTRON type chamber at a temperature of 10 ± 1 °C. The pure microbial biomass was obtained via centrifugation at 10 ± 1 °C/4000 rpm/15 min. The initial biomass concentration in fermentation bioreactors was 250 ± 30 mg d.m./dm^3^.

A sweet whey solution, prepared by dissolving 10.0 g of whey powder in 1.0 dm^3^ of tap water, was used as a substrate in the experiments. Before it had been inoculated with the isolated psychrophilic bacterial strains, the whey solution was pasteurized by heating in a water bath at a temperature of 90±2 °C for 30 min, followed by cooling to 10 ± 1 °C. The characteristics of the sweet whey solution used in the study are presented in Table 1.

**Table 1:** Concentrations of contaminants in a sweet whey solution used in the study

Contaminant	Unit	Mean	Standard deviation
COD	[mgO_2_/dm^3^]	10,207	320
BOD_5_	[mgO_2_/dm^3^]	7093	293
TN	[mg TN/dm^3^]	397	41
TP	[mg TP/dm^3^]	90.1	17.5
VFAs	[gCH_3_COOH/dm^3^]	2.7	0.3
pH	-	7.13	0.16

### Research station

The experiments were carried out under dynamic conditions using Bio Flo 310 type flow-through anaerobic bioreactors (New Brunswick). The bioreactors were equipped with a heating-cooling system (a water jacket), ensuring a temperature of 10 ± 1 °C inside the reactors; control sensors of a foam level, gas concentration, and pH, as well as peristaltic pumps for substrate feeding. The technological system was also equipped with a vertical paddle agitator, ensuring the mixing intensity of 100 rpm. The HRT was 20 days. The initial culture was established in 400 cm^3^ of the substrate solution. Afterward, the sweet whey solution dose (40 cm^3^) and the organic substrate load (OLR, 0.2 g COD/dm^3^/day) assumed in the study design were added every second day. Before introducing the assumed dose of the sweet whey solution to the culture, its equivalent amount was sampled from the reactor for chemical analyses. The experiment was continued for 60 days, which allowed for the three-fold complete hydraulic exchange of bioreactor content.

### Methods of microbiological identification

The samples of above-ground water from aquifers were fixed with paraformaldehyde (PFA, pH 7.4) used in the amount needed to ensure the final concentration of 4% in the sample. The samples were allowed to fix at 4 °C for up to 24 h. After fixing, bacteria were placed on white polycarbonate filters (Millipore, type GTTP, with a pore size of 0.2 mm and diameter of 47 mm) using Millipore filtration equipment. Thus, prepared filters were placed on sterile plastic Petri dishes and stored at a temperature of − 20 °C. Afterward, 20 μL of a hybridization buffer (5 M NaCl, 1 M Tris/HCl, formamide, ddH_2_O, 10% SDS) was injected onto each filter together with an appropriate probe (50 ng/μL), and the filter was placed in a 50-cm^3^ Falcon tube. The preparations were then subjected to hybridization at a temperature of 40 °C for 16–18 h (in an incubator by GFL company, Burgwedel, Germany). The excess of unbound probes was removed by rinsing with a probe-free rinsing buffer (5 M NaCl, 1 M Tris/HCl, 0.5 M EDTA, ddH_2_O, 10% SDS) in a water bath (Memmert, Schwabach, Germany) at a temperature of 48 °C for 15 min. At the subsequent stage, the filters were rinsed with distilled water and ethanol, dried, stuck onto microscope slides, and stained for 3 min using 50 μL of a DAPI dye (4,6-diamidino-2-phenylindole) to the final concentration of 1 μg/mL (Porter and Feig 1980). Next, they were carefully flushed with distilled water and rinsed for a few seconds with 80% ethanol to remove the non-specific bond of DAPI dye. The filters were stuck onto microscope slides using autofluorescence-free immersion oils Citifluor (Agar Scientific, Essex, Great Britain) and VectaShield (Vector Laboratories, Burlingame, USA), mixed in a 4:1 ratio. The preparations were observed under immersion in an epifluorescent microscope (BX61, Olympus) using two types of filters for DAPI and Cy3 (indocarbocyanine).

Selected groups of methanogenic bacteria were detected using fluorescent in situ hybridization (FISH), which enables identifying bacterial groups in the *Archaea* domain and determining population numbers of dominant species of methanogenic bacteria. The following probes were used in the study: ARCH 915 (5′-GTGCTC CCCCGCCAATTCCT-3′) for the *Archaea* domain (Stahl and Amann 1991), EUB338 (5′-GCTGCCTCCCGTAGGAGT-3′) for the *Bacteria* domain (Amann et al. 1990), GAM42 with a competitor (5′-GCCTTCCACATCGTTT-3′/5′-GCCTTCCCACATCGTTT-3′) for *Gammaproteobacteria* (Manz et al. 1992), MSMX 860 (5′-GGC TCGCTTACGGCTTCCCT-3′) for the *Methanosarcinaceae* family bacteria (Raskin et al. 1994), MC 1109 (5′-GCAACATAGGGCACGGGTCT-3′) for the *Methanococcaceae* (Raskin et al. 1994), as well as MB 311 (5′-ACCTTGTCTCAGGTTCCATCTCC-3′) (Crocetti et al. 2006), and MG 1200b (5′-CGGATAATTCGGGGCATGCTG-3′) for the *Methanobacteriales* order (Crocetti et al. 2006). The probe NON338 (5′-ACTCCTACGGGAGGCAGC-3′) was used as a control to check for the non-specific binding of oligonucleotide bonds in the samples examined (Wallner et al. 1993). The oligonucleotide probes (Metabion, Martinsried, Germany) were labeled with Cy3 (indocarbocyanine, red signal). The preparations were observed under immersion in an epifluorescent microscope using two types of filters for DAPI and Cy3.

Single colonies were isolated, and their morphological traits were determined (shape, ciliation, Gram staining, production of catalase, and cytochrome oxidase). The tentative identification of the selected strains was carried out using API 20A tests by bioMerieux developed for anaerobic bacteria. The genomic DNA was isolated from 38 isolates obtained from cultures incubated on the Brucella agar and the Brewer Anaerobic agar (Chen and Ronald 1999). The bacteria were identified by sequencing their 16S rDNA using a BigDye Termiantor v3.1 kit in an ABI 3730xL genetic analyzer (Applied Biosystems, Foster City, USA). The 16S rDNA genes were sourced in the PCR reaction acc. to Gillan et al. (1998) using the following primers: 27F (5′-AGAGTTTGATCATGGCTCAG-3′) and 1492R (5′-GGTACC-TTGTTACGACTT-3′).

### Analytical methods

The sweet whey and the samples collected throughout the experiment were analyzed for BOD_5_ using the Oxi-top control system (WTW, Germany), and for COD, TN, and TP contents using a DR 5000 spectrophotometer with an HT 200s mineralizer (Hach–Lange). The samples’ pH was measured with a VWR 1000 L pH meter, whereas the content of volatile fatty acids (VFAs) was determined with the 0-50 Nanocolor photometric method.

The volume of biogas produced was measured using a mass flow meter (Aalborg), which allowed reading out the momentary gas flow rate and was equipped with an adder, which enabled determining the total amount of biogas produced since the onset of the measurement period. To determine the qualitative composition of the gaseous metabolites of anaerobic bacteria, the biogas flowing through the counter was collected into Tedlar sampling bags and subjected to the chromatographic analysis in a GC Agillent 7890 A chromatograph.

### Statistical analysis

The experiments were carried out in three replications. The statistical analysis of the experimental results was conducted using STATISTICA 13.1 PL package. The hypothesis concerning the distribution of each analyzed variable was verified based on the W Shapiro–Wilk’s test. The one-way analysis of variance (ANOVA) was conducted to establish the significance of differences between mean values, whereas the Levene’s test was used to check for the homogeneity of variance in groups. The HSD Tukey’ test was used to determine the significance of differences between the analyzed variables. The significance level of *p* = 0.05 was assumed in all tests.

## Results and discussion

The present study aimed to verify the hypothesis that the use of psychrophilic and psychrotrophic bacteria in the methane fermentation of sweet whey would yield satisfactory technological effects and would, potentially, allow reducing bioreactor heating costs. The methanogenesis based on a high enzymatic activity of psychrophilic microorganisms isolated from natural ecosystems was expected to ensure biogas production as high as in the case of mesophilic fermentation. So far, attempts of the low-temperature conversion of organic matter to biogas have been usually made with mesophilic anaerobic sludge adapted to low temperatures. There are sparse works on the use of methanogenic bacteria isolated from natural ecosystems in this process.

Investigations on the low-temperature fermentation of organic matter have been carried out as early as the 1990s (Wellinger and Kaufmann 1982; Sutter and Wellinger 1988; Wellinger and Sutter 1988; Zeeman et al. 1988; Wellinger 1989). The researchers proved the necessity to adapt the anaerobic sludge to temperatures ranging from 5 to 10 °C and to use a long HRT (Sutter and Wellinger 1985). Despite attempts to adapt the sludge to low temperatures, gas production increased linearly with increasing temperature (Sutter and Wellinger 1988). The adapted anaerobic sludge was used for biogas production from pig and cattle manure at temperatures ranging from 15 to 20 °C (Nozhevnikova et al. 1999) and for the fermentation of bottom sediments from eutrophicated inland reservoirs rich in organic matter (Nozhevnikova et al. 2007). The results obtained were not promising. The main technological problems included the necessity of ensuring a long HRT and a significant decrease in pH due to the accumulation of volatile fatty acids (VFA). These phenomena were reported to reduce the efficiency of biogas and methane production in the fermentation process run with mesophilic fermentation bacteria adapted to grow at temperatures of 5 to 10 °C (Kashyap et al. 2003). Similar observations were confirmed by Enright et al. (2005) who used the anaerobic treatment to the waste from the pharmaceutical industry. However, significant advances were noted in later studies, especially with regard to pig and cattle manure. Inocula sampled from psychrophilic and mesophilic environments were introduced and their effects on psychrophilic dry anaerobic digestion of cow dung for methane production at 15 °C were investigated in single-stage batch reactors for 84 days. The results showed that the specific methane yield and volatile-solid removal in the fermentation system inoculated with psychrotroph flora had been enhanced by 28.3% and 28.6%, respectively, compared to a system inoculated with mesophilic flora (Zhu and Jha 2013). Gunnigle et al. (2015) in order to investigate the AD microbiome response to temperature change, with particular emphasis on methanogenic archaea, duplicate laboratory-scale AD bioreactors were operated at 37 °C followed by a temperature drop to 15 °C. A volatile fatty acid-based wastewater was used to provide substrates representing the later stages of AD. Methanomicrobiales abundance increased at low temperature, which correlated with an increased contribution of CH_4_ production from hydrogenotrophic methanogenesis at 15 °C. Overall, changes in microbial community structure and function were found to underpin the adaptation of mesophilic sludge to psychrophilic AD. Promising results were also obtained by Martí-Herrero et al. (2015). The aim of their research is to evaluate the co-digestion of cow and llama manure combined with sheep manure, in psychrophilic conditions and real field low cost tubular digesters adapted to cold climate. After 100 days, biogas production was stable, as was the methane content and the pH of the effluent.

**Fig. 1:**
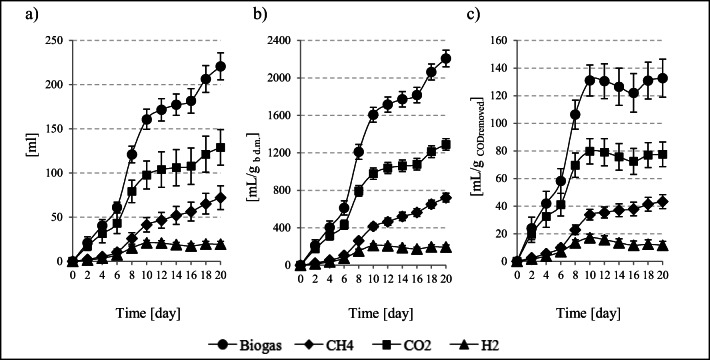
Production of biogas and its main components: **a** cumulative, **b** unitary per g_bd.m._, and **c** unitary per g_CODremoved_

**Fig. 2:**
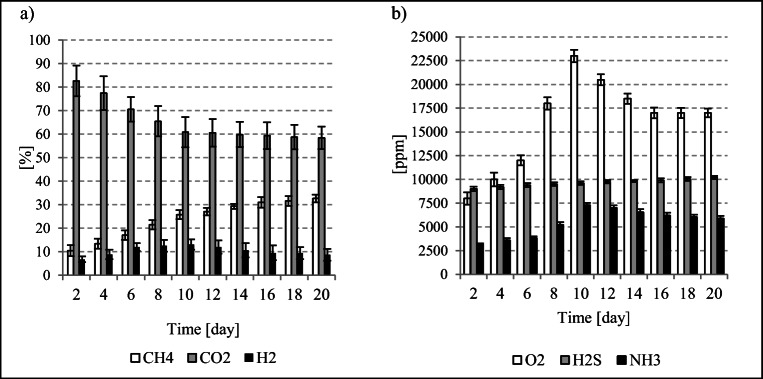
Contents of **a** CH_4_, CO_2_, H_2_ and **b** O_2_, H_2_S, NH_3_ in biogas

Such exploitation and technological problems can be eliminated through the use of strains of typical anaerobic psychrophiles and psychrotrophs (Coté et al. 2006). This hypothesis has prompted the research presented in this manuscript. The total volume of gas produced was at 220.7 ± 15.3 mL (Fig. 1a), whereas gas production reached 2207.2 ± 88.1 mL/g_bd.m._ relative to the dry matter content of the psychrophilic bacteria inoculum (Fig. 1b) and 132.7 ± 13.8 mL biogas/g_CODremoved_ (Fig. 1c). The CH_4_ concentration in the biogas was at 32.7 ± 1.6% (Fig. 2a). The cumulative production of this fermentation gas component was at 72.1 ± 13.5 mL CH_4_ (Fig. 1a), and—when expressed per the initial concentration of bacterial biomass—at 720.9 ± 47.4 mL CH_4_/g_bd.m._ (Fig. 1b). The H_2_ concentration reached 8.7 ± 4.7% (Fig. 2a), which allowed for its cumulative yield of 19.3 ± 3.2 mL H_2_ (Fig. 1a) and 192.5 ± 22.5 mL H_2_/g_bd.m._ (Fig. 1b). The percentage content of CO_2_ in the biogas was at 58.42 ± 2.47% (Fig. 2a). Other gases were also detected, though in lower concentrations, including 17,000 ppm O_2_, 10,200 ppm H_2_S, and 5910 ppm NH_3_ (Fig. 2b).

**Fig. 3:**
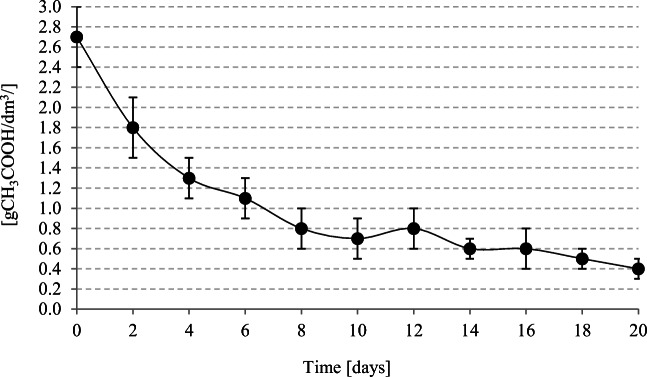
Changes in VFA concentration in bioreactors

A serious exploitation and technological problem signalized by most authors investigating the low-temperature methane fermentation process is the necessity of ensuring a long HRT (Nozhevnikova et al. 2007; Wellinger 1989). The HRT used in the present study was 20 days, because the intended fermentation results were expected to correspond to these achieved under standard technological parameters typical of the facilities operating in the technical scale. Singh et al. (1995) examined the impact of HRT on biogas production under psychrophilic temperatures and demonstrated vast differences in VFAs accumulation at various HRTs. Under conditions of psychrophilic fermentation, a too short HRT accompanied by a high OLR can lead to VFAs accumulation and toxicity to methanogenic bacteria. Ranade et al. (1987) have proved propionate to be toxic to methanogens. When coupled with pH decline, this toxicity can lead to a lower gas production on day 20 of HRT. A short HRT was also reported to inhibit the conversion of higher fatty acids to acetate, i.e., a direct substrate of methanogens. In contrast, a comparative analysis of biogas production from bovine manure at various HRTs and a temperature of 18 °C demonstrated a decreased biogas production with a longer HRT (Bardiya and Chaudhari 2000). In the present study, the initial VFA concentration in the fermentation tank was 2.7 ± 0.3 gCH_3_COOH/dm^3^, and no VFA accumulation was observed throughout the process. The VFA content decreased from 1.8 ± 0.3 gCH_3_COOH/dm^3^ on day 2 of fermentation to 0.4 ± 0.1 gCH_3_COOH/dm^3^ on day 20 of fermentation (Fig. 3), which indicates that the 20-day HRT and a COD load of 0.2 g COD/dm^3^/day were appropriate.

**Fig. 4:**
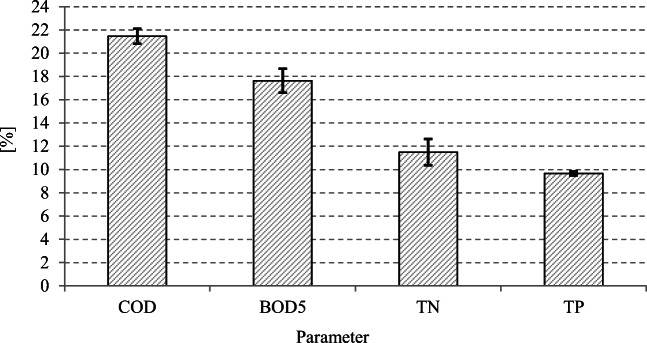
Removal efficiency of organic and biogenic compounds

**Fig. 5:**
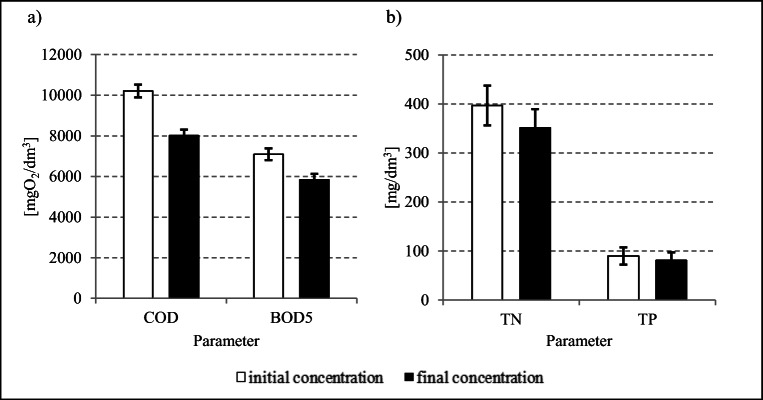
Initial and final concentrations of **a** organic compounds and **b** biogenic compounds

Throughout the experimental period, the COD concentration decreased by 21.4 ± 0.6% (Fig. 4), i.e., from 10,207 ± 320 mgO_2_/dm^3^ to 8018 ± 286 mgO_2_/dm^3^ (Figs. 4 and 5a). In turn, BOD_5_ concentration decreased from 7094 ± 293 mgO_2_/dm^3^ in raw whey to 5844 ± 279 mgO_2_/dm^3^ in the substrate after methane fermentation (Fig. 5a), and the mean efficiency of its removal reached 17.6 ± 1.0% (Fig. 4). The total nitrogen concentration decreased by 11.5 ± 1.1% (Fig. 4), i.e., from 397 ± 41 mg TN/dm^3^ to 351 ± 38 mg TN/dm^3^ (Fig. 5b). In turn, the total phosphorus concentration decreased from 90 ± 17 mg TP/dm^3^ to 81 ± 16 mg TP/dm^3^ (Fig. 5b), and the efficiency of its removal reached 9.7 ± 0.2% (Fig. 4). The pH values recorded were stable and ranged from 7.13 ± 0.26 to 7.19 ± 0.28.

A higher efficiency of COD removal from whey under anaerobic conditions was achieved by McHugh et al. (2006). At the OLR of 0.5–1.3 gCOD/m^3^/day, the efficiency of COD removal at 20 °C was 70–80%. Decreasing the working temperature to 12 °C reduced the efficiency to 50%. The higher COD removal efficiency obtained by these authors was due to a significantly higher sludge concentration in the bioreactors than in the present study, which was conducted using an anaerobic pelleted sludge adapted to psychrophilic conditions. The above authors also proved that decreasing process temperature to 12 °C caused a breakdown of the pelleted sludge’s structure (McHugh et al. 2006). The results presented above are higher than those reported from similar studies on the low-temperature anaerobic treatment of dairy wastewater and whey, but comparable to those achieved under mesophilic (Dugba and Zhang 1999; Gavala et al. 1999) and submesophilic conditions (20–30 °C) (Kalyuzhnyi et al. 1997).

The higher or comparable efficiency of low-temperature methane fermentation is explained by a substantially higher secretion of enzymes by psychrophilic bacteria isolated from natural ecosystems. In many cases, this ability compensates for their lower activity (Russel 2000; Franzmann et al. 1997; Kashyap et al. 2003). The enzymes secreted by psychrophilic and psychrotrophic microorganisms differ from those produced by mesophiles in the lower temperature of their optimal activity, greater thermal stability, and lower activation energy required for substrate hydrolysis (Matthews et al. 2000). Many authors have demonstrated the activity of methanogenic microorganisms in a frozen ground even at a temperature of − 15 °C (Steven et al. 2007a; Steven et al. 2007b; Gilichinsky et al. 2007). In turn, Nozhevnikova et al. (1999, 2001) reported the highest enzymatic activity at low temperatures in the case of bacteria incubated at 4–8 °C. Bacteria of *Methanococcoides burtonii* and *Methanogenium frigidum* species belonging to this domain were isolated from bottom sediments of Antarctic lakes. Their minimal and optimal growth temperatures were at *T*_min._ − 2.5 and *T*_opt._ 23 °C as well as *T*_min._ − 10 and *T*_opt._ 15 °C, respectively (Franzmann et al. 1997; Saunders et al. 2003).

It should also be emphasized that, like in all enzymatic processes, the medium pH plays a significant role in the methanogenesis (Xu et al. 2020). Optimal for the methane fermentation is a slightly alkaline medium; however, the methanogenesis is also effective in a slightly acidified medium (to pH 6.5). In the present study, the pH value in the reactor was very stable and ranged from 6.92 to 7.29.

## Conclusions

The experiments performed confirmed the feasibility of exploiting psychrophilic microorganisms in the methane fermentation of sweet whey. The study yielded 132.7 ± 13.8 mL biogas/g_CODremoved_. The CH_4_ concentration in the biogas was 32.7 ± 1.6%, that of H_2_ was 8.7 ± 4.7%, whereas that of CO_2_ reached 58.42 ± 2.47%. The COD removal efficiency reached 21.4 ± 0.6%. The absolute amount of biogas produced, expressed per organic substrate load, was relatively low compared to the values achieved during mesophilic fermentation. However, relative to microbial biomass concentration in the fermentation tanks tested, it did not diverge from the values obtained in the systems exploited at higher temperatures. Achieving high degradation of organic matter and satisfactory final technological effects requires a correspondingly higher concentration of the bacterial biomass in the system.

It should also be emphasized that the possibility of implementing solutions of this type is currently hindered by many factors related directly to technical difficulties and economic concerns. The major factors which impede the implementation of the described technology include difficulties with sourcing, isolating, proliferating, and ensuring purity of cultures during the exploitation of anaerobic bioreactors. To maintain a pure culture that will assure the expected and stable final effects in terms of the quantity and quality of biogas produced, it is necessary to provide efficient and effective hygienization of substrates and to introduce elements ensuring microbial sterility into the system. However, these treatments increase exploitation and investment costs and pose technological difficulties. However, it seems that these difficulties can be overcome due to the technical advance.

The analysis of the study results does not allow for the explicit and accurate determination of the technological parameters of the process and for the selection of appropriate facilities of the system operating in the full, technical scale. The development of the technical and technological concept of a system for organic substrate conversion into a high-energy biogas should be preceded by gathering more data from the system operating in the fractional-technical scale.

## Data Availability

All data generated or analyzed during this study are included in this published article.
